# Students' Perspectives on Learning Practical Nursing Skills: A Focus Group Study in Norway

**DOI:** 10.1155/2021/8870394

**Published:** 2021-04-09

**Authors:** A. G. Gregersen, M. T. Hansen, S. E. A. Brynhildsen, V. A. Grøndahl, A. C. Leonardsen

**Affiliations:** ^1^Department of Health and Welfare, Ostfold University College, Postal Box Code 700, 1757 Halden, Norway; ^2^Department of Surgery, Ostfold Hospital Trust, Postal Box Code 300, 1714 Grålum, Norway

## Abstract

Practical nursing skills are complex and involve technical, theoretical, and practical aspects, caring perspectives adjusted to both patient and circumstances, as well as ethical and moral considerations. Patients' length of stay in hospitals is decreasing, and more advanced patient treatment is conducted in primary healthcare settings. Hence, education and nursing skills need adjustment in line with the rapidly evolving field of practice. Studies emphasize a need to uncover whether the technical aspect of nursing skills, in general, is challenging in students' learning. The aim of this study was to explore students' perspectives on practical nursing skills and how they can best learn these. Three focus group interviews were conducted with registered nurse students and intellectual disability nurse students in their last semester (*n* = 11). Conventional, inductive content analysis in line with recommendations from Hsieh and Shannon was used to analyze the data. Two main categories with subcategories were identified: (1) the content of practical skills, with subcategories (a) human-to-human relations, (b) organizational competence, and (c) technical mastering and (2) building competence, with subcategories (a) need for supervision, (b) planning the learning situations, and (c) relevance for practice. Students experienced that practical skills did not only include technical aspects but also the ability to establish a relationship to the patient and to organize their working day. Supervising was assumed as essential both when training in the simulation center and in clinical placement, as well as planning of the training, respectively. Students experienced that some skills learned in the university college were less relevant in clinical practice and that certain skills were difficult to perform in practice due to the type of clinical placement. Hence, there is a need to review the approach to and content of practical nursing skills' learning in healthcare undergraduate programs, to prepare students for clinical practice, and to ensure that they build the competence needed in healthcare services.

## 1. Introduction

As a consequence of increasing demands due to the demographic development, with an increasing number of older people and people with chronic illness, the organization of healthcare services and nursing competence needs are changing. Patients' length of stay in hospitals is decreasing, leading to more advanced patient treatment being conducted in primary healthcare settings. Hence, nurse education and nurses' competence need adjustment in line with the rapidly evolving field of practice [1–3].

To ensure quality in patient care, healthcare personnel must be qualified in practical procedure performance [4]. Research indicates that newly qualified nurses experience the nursing demands as complex and overwhelming. They wish for higher competence in concrete situations and better knowledge about procedures they are expected to master [5–7]. Consequently, it has been emphasized that the nurse education curriculum needs to be better oriented towards the healthcare service needs and include more practical procedure training [4, 6]. The performance of practical procedures is complex and involves technical and theoretical aspects, caring perspectives adjusted to both patient and circumstances, as well as ethical and moral considerations [8].

It has been claimed that education of nurses should focus more on factors that influence students' practical skills' learning [9]. Performing practical skills on actual patients is assumed to be more efficient to reach an in-depth understanding than what students achieve through simulation or training in skill centres [10, 11]. Clinical placement is therefore considered a very important learning environment for the development of practical skill competence [8, 12]. Clinical placement is a major component of the nursing education curriculum but provides nursing students with varied opportunities to practice practical skills due to a high degree of specialization and the introduction of innovative medical technologies in healthcare services [13, 14]. Hence, which students have an opportunity to learn depends on where they have their clinical placement. Moreover, supervisors in the clinical placements experience challenges with balancing the responsibility for both patients and the student. In addition, they have limited time to supervise, and they request closer collaboration with the educational institution [15, 16]. Both supervisors and students experience a tension between theory and practice [17, 18]. As a consequence, it has been emphasized that policymakers should focus on improving the clinical environment, enabling for the professional development of students [19].

In Norway, two different bachelor programmes for authorized healthcare personnel with a defined medical competence exist: one for registered nurses (RNs) and one for intellectual disability nurses (IDNs). The defined medical competence for IDNs is related to patients with intellectual and/or physical disabilities, as well as patients with psychiatric illness or addiction. RNs' medical competence is related to patients in primary and specialist healthcare services with primarily somatic and psychiatric diseases. Practical nursing skills' learning across these undergraduate programmes is very much similar and includes both theory and practical skills' training in simulation centres and in clinical placement. Fifty percent of the RN education in Norway consists of supervised clinical placements, while it comprises thirty percent in the IDN education. As both RNs and IDNs are authorized healthcare personnel with competence in practical nursing skills, calls for positions often include both, especially in primary healthcare services.

A recent study found that supervisors perceive that students should learn most practical skills in the educational institution, while they should get further training in these skills when in clinical placements [20]. This may have an impact on supervisors' approach to, or facilitation of, students' learning of practical skills. A review of the literature concludes that the teaching of practical skills is a shared responsibility between nursing education at university-based settings and the training of nursing students during clinical practice [21]. Still, little is known about the ways in which students learn practical skills during their clinical placements [22]. A few studies have been conducted on RN students' learning and performance of peripheral vein cannulation both in university-based and in clinical settings [8, 11, 23]. One conclusion was that low-fidelity simulation was effective, providing familiarity with equipment used in the clinical setting, but also inadequate due to lacking opportunity to discern differences encountered in the clinical setting [11]. A need has been emphasized to uncover whether the technical aspect is challenging in students' learning of practical nursing skills in general [8]. Hence, researchers emphasize the need to explore RN students' learning and performance of technical aspects of other practical nursing skills [11, 24]. We are unable to identify research on IDN students' learning of practical nursing skills.

Consequently, the aim of this study was to explore RN and IDN students' perspectives on practical nursing skills and how they can best learn these.

## 2. Materials and Methods

A qualitative, explorative design was used. A focus group is an interview technique that uses purposive sampling to select participants, who are of a specific population, share similar characteristics, and have something to say about the topic [25]. Focus groups are appropriate when the aim is to explore areas that need improvement based on participants' perspectives and ideas [26, 27]. The participants' experiences are deepened and developed through discussions and dialogue between the participants [28]. Hence, this method was assumed appropriate for the aim of this study.

The authors consist of RN and IDN student educators (*n* = 5). The authors are also part of a research group that consists of an IDN working in the university college simulation center, three RNs working in a hospital, and three RNs working in primary healthcare services. The research group was included in the planning of the study, as well as in interpretation of the findings. The manuscript adheres to the Standards for Reporting Qualitative Research (SRQR) [29].

### 2.1. Study Setting

The university college is located in an area that covers 320,000 inhabitants. The clinical placement of students takes place in a hospital with two different geographical locations: one with elective services only and one with both acute and elective services. Primary healthcare services include, e.g., acute care wards, casualties, nursing homes, homes for people with intellectual or functional disabilities, and home-based nursing services. The students have six (RN) and three (IDN) periods of clinical placement, respectively, in different wards and healthcare levels, during a three-year undergraduate program. They receive theory and training in practical skills in the simulation center during their first and second year of education (RN students), and IDN students in the third year as well. Practical skills' learning includes many different skills, varying from simple to complex skills, and from, e.g., bed making to catheterization. Cardiopulmonary resuscitation is trained all three years in both programs.

### 2.2. Participants

We chose to include RN and IDN students in their last semester of education. As of 2020, this included 162 RN students and 59 IDN students. A purposive sampling method was used. All students from each educational program were invited to participate through e-mail. In total, 11 students responded and were consequently included.

### 2.3. Interview Guide

An interview guide was developed based on earlier research [15, 16, 18, 30] and informal feedback from supervisors in primary and specialist healthcare services, as well as several discussions between the participants in the research group, until consensus was reached. Feedback indicated that the guide was concrete, relevant, and understandable. The guide consisted of five different themes: practical skills, preparedness, self-assessed competence, mastering, and the educational program (see Supplement 1).

### 2.4. Procedure

The focus group interviews were conducted in a meeting room at the university college and lasted from 40 to 55 minutes. The focus groups were led by a moderator and an assistant moderator. We ensured that the RN educator participated in the IDN student interviews, and vice versa. The assistant moderators were two RNs not working in the university college. The assistant moderator observed the interaction in the group and noted down observations and nonverbal communication. The moderator focused on letting the participants freely discuss their experiences related to the themes presented. Participants were encouraged to exchange experiences and spontaneously comment on each others' views and statements. The interview guide was used as a support to ensure that all themes were covered in both the focus groups and the interview with two participants.

The interviews were digitally recorded. All records were transcribed verbatim by an external transcriber, who had signed a nondisclosure agreement. The recordings were deleted after transcription.

### 2.5. Ethical Considerations

The study was conducted in line with recommendations in the Declaration of Helsinki [31]. Students received oral and written information about the study purpose and delivered signed written consent to participate. Due to the nature of a focus group, it was not possible to withdraw from the study. Participation was voluntary. The study was approved by the Norwegian Center for Research Data (NSD, reference no: 95194). All the data were handled confidentially. It is not possible to recognize individuals in the transcripts or in the presentation of results. To ensure anonymity, students were given codes/numbers: RN 1–5 and IDN 1–6, respectively.

### 2.6. Analysis

We used a conventional, inductive content analysis in line with recommendations from Hsieh and Shannon [32]. The analysis followed four steps: (1) reading and rereading the transcripts to get an overall impression of the data (AGG, MTH, and ACL); (2) identification of keywords and meaningful units (coding): this included making notes of first impressions, thoughts, and initial analysis. Labels for codes emerged that were reflective of more than one key thought (the initial coding scheme) (AGG and MTH); (3) codes were then sorted into categories based on how different codes were related and linked. These emergent categories were used to organize group codes into meaningful clusters (AGG, MTH, and ACL). These were then presented and discussed between all authors; and (4) development of definitions for each category/subcategory, where examples for each category were identified from the data to prepare for reporting the findings.

In addition, a reflexive method was used to raise awareness among the researchers on factors that could have affected the interview process and dynamics [33]. Directly after each interview, the researchers noted down initial impressions and thoughts from the interview. The notes focused on student activity, own thoughts, and own experiences from many years of experience as educators and supervisors in clinical placement. This was included and discussed during the analysis process.

During the analysis, the transcripts were included in a table. Keywords were marked yellow. Meaningful units were then transferred to the next column (initial coding scheme), and collated categories were placed in the next column. This was an iterative process, moving back and forth from transcripts to codes to categories. The analysis consisted of several discussions between the researchers until consensus was reached. See Table 1 for an example of the analysis process.

## 3. Results

Three focus group interviews comprising five RN students (1 male) and six IDN students (2 males) were conducted in the period October to December 2018. The age range of the participants was from 25 to 35 years.  Table 2 gives an overview of participants in different focus group interviews.

Through analysis, two main categories with subcategories were identified: (1) the content of practical skills, with subcategories (a) human-to-human relations, (b) organizational competence, and (c) technical mastering and (2) building competence, with subcategories (a) need for supervision, (b) planning the learning situations, and (c) relevance for practice. The difference between RN and IDN students was that IDN students talked more about competence in communication related to aggressive patients and clients. Otherwise, practical skills were described very much similarly by all participants.

## 4. The Content of Practical Skills

### 4.1. Human-to-Human Relations

All students experienced a need for competence in collaboration and communication and defined this as practical skills. In clinical placement, students found it necessary to be able to meet people in different situations. This was described as to “tune in on,” “be aware,” and “be sensitive” to the patients' situation and condition. They gave several examples of this, e.g., one of the IDN students described especially challenging situations:

…aggressive actions, and how to treat people humanely, and at the same time limit their behavior (IDN 3)

The students found it essential that they were able to treat the patient with dignity in such situations. Moreover, they were concerned about how to handle interaction appropriately, for the patients' as well as for their own sake. Students talked about being sensitive to the patients' vulnerability, for example, in care situations. One of the RN students stated that

Care situations, it is a very vulnerable situation for the patient, and that is why we have to know something about that. Something I have learned from the skill training in school is how to act within the circle of intimacy (RN 2)

This was verified by nodding in the rest of the focus group.

Students emphasized that nurses always have to pay attention to the patient and that patients' needs always are in focus. This was also interpreted as an observation, giving an opportunity to adjust nursing practice accordingly. One of the IDN students prompted

We continuously communicate with the patient, observing facial expressions all the time, we don't do anything without… we're not blind when we are there, we continuously adjust and change according to the patients' needs (IDN 1)

### 4.2. Organizational Competence

To the IDN students, the practice field seemed complex and requiring different kinds of competence. They talked about being able to guide clients, patients, and relatives in meeting different health and social services. One of the IDN students said that

We need knowledge on how to guide the client through a quite complex system (IDN 4)

Organizational competence was described as “to know the organization,” “be able to plan,” and “to have an overview.” One way to show this was to know the distribution of duties during both day and night shifts. One of the RN students said that

I find it important to know who is meant to do what in daytime, evening, night… Then I have the overview… (RN 1)

Organizational competence also included being effective. One of the RN students prompted

… to be able to plan the actions without much extra work (RN 5)

Moreover, organization was also related to planning of collaboration between professionals. One of the IDN students gave an example of this:

… and during the doctors' visits, if you're not updated on patients' somatic health, then you cannot reach far … both regarding procedures and tests before the visit starts, right … (IDN 1)

### 4.3. Technical Mastering

All of the students talked about several practical procedures they needed to know. These varied from basic practical skills such as making a bed to procedures such as vein cannulation or blood sampling. Students claimed that they gained a basis for technical mastering when training in the simulation center. Moreover, they experienced that training in specific procedures was generalizable to other procedures, e.g., related to aseptic principles. One of the RN students said that

… many procedures need to be performed aseptically, for example catheter insertion or wound care…To know aseptic principles is essential … (RN 4)

The IDN students more clearly described experiencing, not mastering, the same skills as RN students, even though they experienced the same relevance of these procedures in clinical placements. This was, for example, related to vein cannulation and blood sampling.

Mastering was experienced as a result of the combination of theory, training in school, and training in clinical placement. The IDN students experienced not getting the same preparedness in school as the RN students, even though they needed this competence in clinical placement. One of the IDN students described this:

… for example related to handling medications. Of course, I could read about the drug, but still there are some basics you need to know … Can you give him this dose of paracetamol in relation to ibuprofen? There are several interactions with the most common drugs … We have not received any lectures or training in this, as far as I can recall … (IDN 2)

The rest of the focus group expressed agreement with this.

The students experienced mastering after clinical placement that gave them the opportunity to repeat practical skills. This was especially related to clinical placement in hospitals. One RN student said that

It is mass-training, because you do it many times during a shift (RN 3)

One of the other RN students continued

In the surgical ward we have done a lot of wound care. I would not say that I am 100 percent secure, but I feel that I know a lot about this … And I have also been in an observation ward, taking ECG daily. So, I feel secure in taking an ECG, but not in how to read it (RN 2)

## 5. Building Competence

### 5.1. Need for Supervision

The students had various experiences with supervision, and they wanted more supervision and different sorts of supervision. This was expressed differently, but all of the students in all of the three focus groups described “to be shown,” “to be drilled,” and “to be pointed out” as different approaches to supervision. All of them emphasized that healthcare services are hectic and that there is not always room for the supervision they need. In the simulation center, they experienced having more time and supervision available. At the same time, they emphasized that a teacher had to be present during practical skills' training to secure the quality before students practice on real patients.

In one of the focus groups, students stated that it was embarrassing when they did not know the skills and procedures before clinical placement. They wanted to be prepared to avoid discomfort or pain. One of the RN students stated that

You can feel insecure yourself, but to be able to perform without the patient feeling this … (RN 4)

To be able to do so, students reported the need of a supervisor present in the situation and the need for concrete guidance and feedback. One of the IDN students said that

One-to-one, I would say. It is best to be together with your supervisor in practice, and then he or she does it, and then you do it…While that person watches and tells if you are doing it right. That is a good way to make you feel safe, yet able to try out things (IDN 6)

### 5.2. Planning the Learning Situation

The university college requests a plan from the student for the whole placement period as a pedagogical tool. The students had ambivalent experiences regarding this. Several of both RN and IDN students found this disturbing during clinical placement. One IDN student said that

It takes the focus away from other things that perhaps is more important (IDN 1)

Some students reported not understanding the purpose of the plan at first, but after a while, they thought the plan was useful. One of the RN students prompted

Then you have something to work with, an agenda you should have learned throughout your placement. In addition to all, I believe it worked (RN 4)

Another advantage was that the plan committed and ensured quality at the placement ward. Several students had experienced that the supervisors' colleagues at the placement ward helped to look for learning situations according to the plan. Often, the situation was more important than following their supervisor, and the plan then made this possible.

### 5.3. Relevance for Practice

The students wanted more relevant clinical placement arenas and also more clinical practice. After training in the simulation center, several of them had not had an opportunity to train clinical skills on real patients. Moreover, the IDN students experienced that the periods between clinical periods were too long and that this resulted in insecurity for the students' own achievements regarding their learned practical skills. In addition, all of the students reported that they were not given the opportunity to try out things they had learned at the university college in clinical placement, even though they found it relevant, such as the IDN students' experience of behavioral therapy. One of the IDN students expressed that

It is a basic thing, but is not a priority. I feel that I work in places where it could have been relevant, but it doesn't happen… Then they got people from the outside to do it (IDN 1)

All of the students had met procedures and practical skills in clinical placement that they had not learned in the university college. For example, one of the IDN students was given the responsibility to perform exercises with a patient with cerebral paresis and talked about this experience:

I was supposed to do exercises with this patient. But how do I do that? It means to train someone who is completely stiff, and then a nurse came and tried to show me how to do it…We have never learned about that … (IDN 3)

Another issue was that students experienced that techniques that were highlighted at the university college were not relevant in clinical placement, e.g., one of the RN students said that

Because, if you're supposed to wash someone the way we learned in the simulation center, we would not have been finished in eight hours … It is not the way it is done. That is not how it is being practiced, so that in itself is interesting”. … (RN 3)

## 6. Discussion

The aim of this study was to explore IDN and RN students' perspectives on practical nursing skills and how they can best learn these skills. Our findings show that students associated practical skills with the establishment of human-to-human relations, organizational competence, and technical mastering of practical procedures. To learn practical skills, students talked about building competence. Here, they emphasized the need to be supervised, the importance of planning the learning situations, and the importance of being able to train on skills in clinical placement and learning relevant practical skills in the university college.

Competence in building human-to-human relations was described by both RN and IDN students as an essential skill. In 1971, Joyce Travelbee developed the “Human-to-Human Relationship Model.” [34] She believed nursing is accomplished through human-to-human relationships that begin with the original encounter and then progress through stages of emerging identities, developing feelings of empathy, and later feelings of sympathy [35]. Travelbee's model provides nurses with a foundation necessary to connect therapeutically with other human beings [36]. Globally, there are a relatively small number of studies dealing with this issue.

The importance of achieving a mutual understanding in creating interpersonal relationships, communication skills of nurses, and overcoming of nurse-patient stereotypes has been emphasized to be able to provide safe and quality healthcare services [37]. Additionally, in recent years, there has been an increasing focus on patient-centredness in healthcare. Ekman et al. distinguished between patient‐centred care and person-centred care by which person-centred care refrains from reducing the person to just their symptoms and/or disease [38]. Conceptually, person-centred care is a model in which healthcare providers are encouraged to partner with patients to codesign and deliver personalized care. This provides people with high‐quality care they need and also improves healthcare system efficiency and effectiveness [39]. Hence, this study shows that human-to-human relation building, or person-centred care, is an overarching concept also for students and is defined as a practical skill that needs to be learned.

Nurses need to perform different tasks during the course of day and to cope with time limitations and pressure. Good time management leads to greater productivity, less stress, improved efficiency, and more opportunities for professional advancement [40]. Students in our study emphasized the need for such organizational skills, which have also been emphasized as important in several studies [41–44].

Students in our study emphasized the need of both theory and simulation training and training on actual patients as important when learning to perform different practical skills. Ravik et al. requested more studies on technical aspects of nursing skills' learning [11, 23, 24]. Moreover, Ravik et al. distinguished between students “knowing that” and “knowing how” as a framework to guide development and competence in the practical skill vein cannulation. The researchers found that practicing the skill on a mannequin and on actual patients gave different learning opportunities. They concluded that low-fidelity simulation provides familiarity with equipment used in the clinical setting but that it is inadequate due to lacking opportunity to discern differences in clinical settings [11]. In 2000, Howanitz et al. [45] outlined four levels of competence: (1) what an individual ‘‘knows” measured by his or her general knowledge, (2) if an individual ‘‘knows how” to act, measured by his or her competence level, (3) if an individual ‘‘shows how” to act, as measured by his or her performance, and (4) what an individual “does,” as measured by his or her action. Referred to our findings, students often “know” and “know how,” but seldom get to “show how” or “do.”

Both RN and IDN students emphasized the need for supervision, both in clinical placement and when training in the simulation center. This is in line with findings in a study, where students report that they seek, lack, and crave more instructions concerning what and how to learn clinical skill procedures [46]. A review of the literature from 2016 also showed that supervisory relationships, peer relationships, and clinical education structure had an impact on nursing students' learning of practical skills [47]. Researchers claim that nursing education must reexamine current methods to practical skill learning, to enhance supervisory relationships and the pedagogical atmosphere, and seek methods to better prepare future nurses [48–50]. Our findings indicate a need to review the education curriculum to increase the relevance in clinical practice. This is supported by studies indicating that nursing students report that the exercises in the university are a good way to prepare for clinical placement but that this does not resemble how it is conducted in clinical practice [51–53]. In addition, a lack of relevance makes students feel unprepared, and the responsibility is overwhelming when facing “reality.” [51–53]

The importance of planning the clinical placement period, with using a plan, is supported by, e.g., Helgesen et al., who showed that students use the extra time filling out the plan reflecting on the procedures they had been observing. Through this reflection, students were able to focus less on technical aspects and more on the patient [54]. Planning also leaves more of the responsibility on learning on the students themselves and is not totally dependant on the supervisor-educator-student relationships.

Even though the undergraduate programs are different, both RNs and IDNs are authorized healthcare personnel meeting healthcare services' expectations of competence in practical nursing skills. Provision of learning opportunities, staff support and supervision, and better coherence in how skills are taught in the educational institutions as well as the clinical setting have been shown to promote learning of practical skills [52, 55]. This is in line with findings in the current study. RN students have more clinical practice than IDN students. Nevertheless, they also wanted more practice and more supervision and focus on more relevant practical nursing skills. This supports earlier research, indicating that practical skills should be learned in a clinical setting [18, 19]. Moreover, it supports the importance of clinical placement in addition to simulation and skills' training in simulation centres.

### 6.1. Limitations

One limitation of this study is the inclusion of few participants, and the inclusion of more participants might have provided additional data. In addition, one interview had only two participants. Even though there were a limited number of participants in this interview, rich data were provided. Furthermore, the students in all focus groups provided detailed accounts and also challenged each other's opinion. This indicates a sense of openness among the participants and demonstrates the generation of good-quality data. Of course, we could have chosen to include students from other semesters or another assembling strategy to include participants in the focus groups.

In this study, we included IDN and RN students. Both IDNs and RNs are authorized healthcare personnel with medical competence. Both groups include practical nursing skills as part of their responsibility in both primary and tertiary healthcare services. Hence, findings are transferable across educational programs, focusing on competence building in practical nursing skills.

The strength is that our findings are in line with recent studies on learning of practical skills and clinical placement in nursing students. Presentation of the analysis and results is transparent, and the researchers used a method of reflexivity to ensure awareness on own preconceptions and how these may have affected the process, which also increases the validity of the findings.

## 7. Conclusions

This study fills a knowledge gap regarding aspects that influence IDN and RN students' learning of practical skills and how they can best learn these. For students, practical skills included human-to-human relations, organizational competence, and technical mastering. When building practical competence, students emphasized the need for supervision and planning of relevant learning situations. Our findings indicate a need to review the educational curriculum comprising practical skills. Moreover, findings indicate a need to improve the collaboration between educational institutions and the clinical field to enhance the quality of practical learning situations for students.

## Figures and Tables

**Table 1:** Example of the analysis process.

Transcripts	Meaningful units (codes)	Categories
No 1: I think about communication… No. 2: To learn how to communicate with different patients, tune in on the patient, you talk different to different persons, children, adults… No. 4: Different conditions they have, be sort of aware, in relation to the situation…be sensitive to that… No. 4: I was allowed to do that in practice, take blood samples, and then I was taught how…But it was kind of…how many times did I try, maybe four patients or so…So you could not say I know how to do it. I have only done it four times… No. 3: You need to repeat it several times…	Communicate and collaborate Be aware of different patients Be sensitive to different situations Allowed to do it in practice Do not learn procedures by doing it only four times Need repetition and further training	Human-to-human relations Need for supervision

**Table 2:** The three interviews.

	Focus group 1	Interview	Focus group 2
RN students (*n* = )	—	2 (1 male)	3
IDN students (*n* = )	6 (2 males)		

RN = registered nurse students. IDN = intellectual disability nurse students.

## Data Availability

The data used to support the findings of this study are available from the corresponding author upon request.
