# Supplementary material for: Students' Perspectives on Learning Practical Nursing Skills: A Focus Group Study in Norway
Source: Nurs Res Pract. 2021 Apr 9;2021:8870394. doi: 10.1155/2021/8870394 (PMC8052176; doi:10.1155/2021/8870394)
Supplement: Supplementary Materials — The interview guide is provided as the supplementary file. [file 8870394.f1.docx]

| **Theme 1. Practical skills** |
| --- |
| What do you think when I say «practical skills»? |
| **Theme 2. Preparedness** |
| How would you evaluate your own preparedness regarding practical skills prior to clinical placement in different wards?  What was the basis for this preparedness? |
| **Theme 3. Self-assessed competence** |
| How would you describe your own self-assessed competence related to practical skills?  Are there areas you feel more competent in?  How did you gain this comptenece?  Which practical skills are most important? |
| **Theme 4. Mastering** |
| What do you think about mastering and practical skills?  Can you describe a concrete situation where you experienced mastering? Or where you did not experienced mastering? |
| **Theme 5. The educational programme** |
| What do you think about the educational programme regarding practical skills in the university college?  Has this met your needs before clinical placement?  If not, what have you missed? |
